# A merged lung cancer transcriptome dataset for clinical predictive modeling

**DOI:** 10.1038/sdata.2018.136

**Published:** 2018-07-24

**Authors:** Su Bin Lim, Swee Jin Tan, Wan-Teck Lim, Chwee Teck Lim

**Affiliations:** 1 NUS Graduate School for Integrative Sciences & Engineering (NGS), National University of Singapore, #05-01, 28 Medical Drive, Singapore 117456, Singapore; 2 Department of Biomedical Engineering, National University of Singapore, 4 Engineering Drive 3, Engineering Block 4, #04-08, Singapore 117583, Singapore; 3 Sysmex Asia Pacific Pte Ltd, 9 Tampines Grande, #06-18, Singapore 528735, Singapore; 4 Division of Medical Oncology, National Cancer Centre Singapore, 11 Hospital Drive, Singapore, 169610 Singapore; 5 Office of Clinical Sciences, Duke-NUS Medical School, 8 College Road, Singapore 169857, Singapore; 6 Institute of Molecular and Cell Biology, A*Star, 61 Biopolis Drive, Proteos, Singapore 138673, Singapore; 7 Mechanobiology Institute, National University of Singapore, #10-01, 5A Engineering Drive 1, Singapore 117411, Singapore; 8 Biomedical Institute for Global Health Research and Technology, National University of Singapore, #14-01, MD6, 14 Medical Drive, Singapore 117599, Singapore

**Keywords:** Non-small-cell lung cancer, Data integration, Gene expression

## Abstract

The Gene Expression Omnibus (GEO) database is an excellent public source of whole transcriptomic profiles of multiple cancers. The main challenge is the limited accessibility of such large-scale genomic data to people without a background in bioinformatics or computer science. This presents difficulties in data analysis, sharing and visualization. Here, we present an integrated bioinformatics pipeline and a normalized dataset that has been preprocessed using a robust statistical methodology; allowing others to perform large-scale meta-analysis, without having to conduct time-consuming data mining and statistical correction. Comprising 1,118 patient-derived samples, the normalized dataset includes primary non-small cell lung cancer (NSCLC) tumors and paired normal lung tissues from ten independent GEO datasets, facilitating differential expression analysis. The data has been merged, normalized, batch effect-corrected and filtered for genes with low variance via multiple open source R packages integrated into our workflow. Overall this dataset (with associated clinical metadata) better represents the diseased population and serves as a powerful tool for early predictive biomarker discovery.

## Background & Summary

The big data boom heralds a new era of precision medicine – access to large pools of ‘omics’ data has driven breakthroughs in this emerging field. In particular, microarray technology is one of the most extensively explored high-throughput methodologies for the quantitative assessment of gene expression^1,2^. The Gene Expression Omnibus (GEO) database at the National Center for Biotechnology Information (NCBI) was launched in 2000 to support public use of such genomic resources provided by the scientific communities^3,4^. Since then, 94,577 series probed with 18,138 platforms, for over 2 million samples have been submitted to the GEO database.

The challenge with these vast datasets, however, is that exploring a huge breadth of data is not straightforward – from effectively querying the correct dataset to utilizing the right pipelines for realizing true significance from such high-dimensional data. Successful differential expression analyses, for example, are reliant on careful interrogation to minimize non-biological variations. Preprocessing of microarray data is thus an essential step prior to downstream analysis. Several preprocessing pipelines exist for background correction and normalization of array-dependent gene expression. The most commonly used techniques are Robust Multiarray Average (RMA)^5^, frozen Robust Multiarray Analysis (fRMA)^6^, Single Channel Array Normalization (SCAN)^7^, and Universal exPression Code (UPC)^8^. The fRMA method was chosen in this study for its use in the *InSilico DB* package^9^ implemented in our developed framework.

The merging of multiple genomic datasets into a single matrix for large-scale meta-analysis poses another source of variation termed the batch effect. Such bias arises as a consequence of systematic technical or non-biological differences between independent laboratories^10^. It is nonetheless possible to adjust this inter-dataset variation with previously established models for such batch effect removal. These include the Empirical Bayes method, also known as ComBat^11^, the Batch mean-centering (BMC)^12^, the Gene standardization (GENENORM)^13^, and the distance-weighted discrimination (DWD)^14^. The Combat method was applied to ten fRMA-preprocessed microarray datasets in this work for the integration into a single dataset.

**Figure 1:**
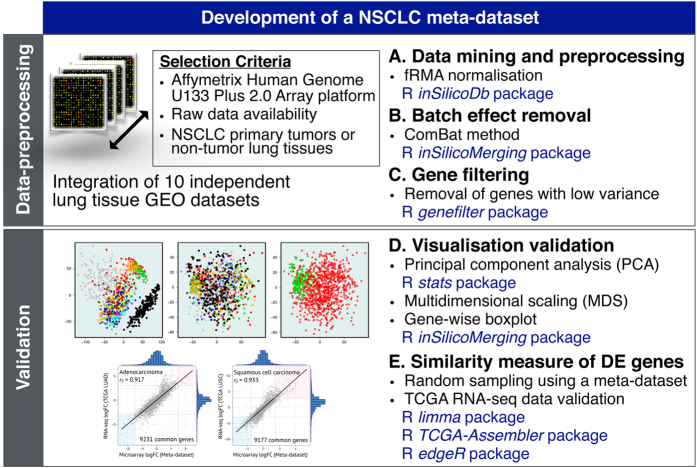
Study design. Preprocessing of raw data from ten independent datasets was done for normalization, background correction and probe-to-gene mapping. The fRMA-normalized data were corrected for batch effect using ComBat method and filtered for genes with low variance across samples. Validation of our dataset was done with PCA analyses and similarity measurement using RNA-Seq-profiled samples. Statistical R packages used to develop this dataset are stated.

Here, we present an integrated R pipeline and a transcriptome dataset for non-small cell lung cancer (NSCLC), together with its associated clinical metadata (Fig. 1). Using this strategy, we recently identified an expression pattern of specific genes that could serve as an accurate clinical tool for its predictive value in prognosis and adjuvant therapy response in NSCLC^15^. Our unique selection and integration of multiple open source R packages greatly reduce computational complexity and processing time to ultimately identify putative cancer-associated gene signatures. To facilitate gene differential expression (DE) analyses, we processed a total of 1,118 patient-derived samples including primary tumors as well as tumor-free control tissues. Additionally, we embedded two robust quality control metrics utilizing RNA-Seq data from the Cancer Genome Atlas (TCGA) in the present pipeline for multi-platform assessment and validation of differentially expressed genes. This normalized dataset serves as an excellent large-scale ‘discovery cohort’ for identification of clinically relevant NSCLC biomarkers.

## Methods

Detailed methods, including the study design and statistical analyses, for constructing NSCLC gene panel and developing clinically applicable risk scoring metrics for patient stratification and prognostication can be found in our recent publication^15^.

### Data collection and preprocessing

The raw data of gene expression profiles from ten independent GEO datasets comprising a total of 1,118 NSCLC samples including both primary tumors and normal lung tissues were downloaded from the NCBI via the *inSilicoDb* package^9^. Samples processed using the same chip platform (Affymetrix Human Genome U133 Plus 2.0 Array) were analyzed (Table 1). This minimizes batch effects that arise from different microarray platforms and allows the analysis of the same set of genes with the same probesets. The fRMA method was first applied to the raw data via the *getDataset* function for background correction, normalization and probe-to-gene mapping. This embedded function allows fast data accession and simultaneous preprocessing of expression profiles, regardless of the screening platform. All clinical information annotated in ten initial datasets were further collected and curated for clinical model development (Data Citation 1).

### Batch effect removal

**Figure 2:**
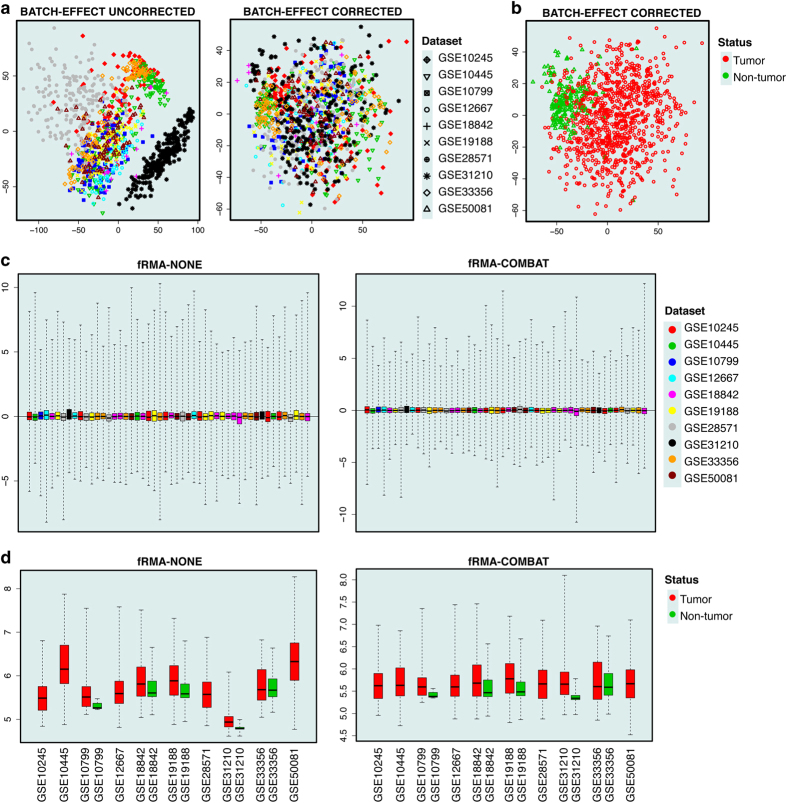
Validity of our generated dataset. (**a**) The effect of batch effect removal is clearly demonstrated using the *plotMDS* function. (**b**) The MDS plot of our merged microarray dataset shows a clear separation between different disease phenotypes (925 primary NSCLC tumors: red; 193 non-tumors: green). (**c**) The merging effect of the ComBat technique on the fRMA-normalized data is illustrated using the *plotRLE* function. (**d**) The local effect of the ComBat method at the gene-level is demonstrated using the *plotGeneWiseBoxPlot* function. *A1BG* gene was selected for the demonstration purpose.

Using the *inSilicoMerging* package^16^, we next merged ten fRMA-preprocessed datasets and corrected for batch effects that arise from technical variation between independent studies. The *merge* function included in this package is simple and straightforward to use for batch effect correction, regardless of the number of independent datasets being queried. Of existing batch effect removal techniques, the ComBat method^11^ was applied to these preprocessed microarray datasets. Technical validation of any chosen method can be done using embedded functions such as *plotMDS*, *plotRLE*, and *plotGeneWiseBoxPlot*. These features allow visual demonstration of reduced variance via the Principal Component Analysis (PCA) approach. Only the first two PCs are plotted as these variables capture the most significant patterns of variation which arises as a consequence of non-biological difference across independent batches^10^. In our recent study^15^, we used the *prcomp* function in the *stats* package and the *ggbiplot* function in the *ggbiplot* package^17^ for generating PCA graphs and subsequent visualization, respectively. In this work, we demonstrate the batch effect removal using the embedded *plotMDS* function (Fig. 2).

### Gene filtering

Genes with low variance across samples can be filtered prior to performing DE analysis. This step prevents flat genes from affecting the downstream analysis and improves the computational processing time by focusing on only statistically significant genes in a meta-analysis. Our integrated dataset stores a huge amount of transcriptomic data, including expression values of 20,155 genes for 1,118 NSCLC patients. Gene filtering was performed using the *nsFilter* function in the *genefilter* package^18^, removing 10,078 genes for subsequent identification of DE genes.

### Code Availability

The R code used to generate our normalized dataset and all the plots described in this paper (and in our recent work^15^) can be found in figshare (Data Citation 1).

## Data Records

Our normalized microarray dataset with associated clinical metadata is available at ArrayExpress (Data Citation 2). DE gene lists with full description are deposited as individual text files in figshare (Data Citation 1). These include annotations of log 2 fold-change, average expressions, t, *P*-value and adjusted *P*-value derived from both microarray and RNA-Seq platforms. All the GEO datasets processed through our pipeline are available from the National Center for Biotechnology Information Gene Expression Omnibus (GEO) databases (Data Citation 3, Data Citation 4, Data Citation 5, Data Citation 6, Data Citation 7, Data Citation 8, Data Citation 9, Data Citation 10, Data Citation 11, Data Citation 12).

## Technical Validation

### Visual validation of batch effect removal

The following functions available in the *inSilicoMerging* package^16^ are used to check the validity of our approach in correcting for batch effects. In this study, the ComBat adjustment is visualized at both systemic and gene-specific levels.

#### A. The *plotMDS* function

The effect of ComBat technique is clearly demonstrated on ten preprocessed datasets (Fig. 2a). The resulting MDS plot in Fig. 2b shows a clear separation of the samples according to the disease phenotype (biological variation), and not the source of dataset (non-biological variation), highlighting successful removal of the batch effect in this merged dataset.

#### B. The *plotRLE* function

Similarly, other functions implemented in the present pipeline can be used to visualize the statistical correction. Here, we randomly selected 50 samples using the RLE plots for demonstration purposes (Fig. 2c). Samples are colored according to the study they are extracted from. Although not as clearly visible as the *plotMDS* function due to large number of variables, the merging effect of ComBat transformation can clearly be indicated using the *plotRLE* function.

#### C. The *plotGeneWiseBoxPlot* function

Unlike the two above-mentioned functions, the last visualization technique included in our R framework shows the local effect of batch effect adjustment at the individual gene level. For demonstration purposes, we selected *A1BG* gene to be illustrated in the gene-wise boxplot (Fig. 2d). A notable change in expression of this gene resulting from the adjustment again demonstrates the validity of the merging technique used in our integrative pipeline for the identification of DE genes.

### Multi-platform assessment of DE genes

The following steps implemented in our workflow aim to address continuing concerns raised in previous works regarding reproducibility of DE genes using the microarray platform^19,20^. Briefly, we first performed random sampling using our generated dataset and derived ranked list of DE genes with each iteration. A significant overlap between ranked lists was indicated by a high overlap coefficient, showing high intra-platform reproducibility in differential gene expression. We further compared DE gene signatures generated from our normalized dataset with that from RNA-seq platform using the TCGA database and observed high inter-platform concordance. Altogether, these additional steps in our pipeline ensure the reproducibility of potential cancer biomarkers derived from our dataset.

#### A. An iterative approach - random sampling

We first determined DE genes using our NSCLC dataset via the *limma* package^21^ by applying the following statistical criteria: (1) log 2 fold change >1.5; (2) adjusted *P*-value<1.0E-10. Such stringent cutoff thresholds produce only a handful of significant genes that distinguish tumors from tumor-free lung tissues. To dispel any possible bias against our feature selection, we performed random sampling using our dataset – the overlap coefficient was computed using all DE gene lists derived from 10,000 iterations. The mean overlap coefficient of 0.899 was obtained in our previous work^15^, validating the robustness of our approach in identifying DE genes. Overall, we show a simple, yet reliable meta-analysis pipeline for discovering reproducible DE genes and facilitating development of clinically applicable models.

#### B. Different profiling platform – TCGA RNA-Seq data

As our dataset exclusively comprised of datasets probed with the same platform (microarray), we further investigated the generalizability of our merged data using RNA-Seq-assayed samples. Level-3 RNAseqV2 gene expression profiles of lung adenocarcinoma (LUAD) and lung squamous cell carcinoma (LUSC) from TCGA were preprocessed via the *TCGA-Assembler* package^22^ for subsequent DE analyses. The raw sequencing data were first normalized with RNA-Seq by expectancy maximization (RSEM) method using the *DownloadRNASeqData* function. Prior to DE analysis, these RSEM-normalized data were preprocessed using the *DGEList* function and only genes expressing at a counts-per-million (CPM) above zero in at least 20% of the samples were retained using the *cpm* function via the *edgeR* package^23^. The resulting data were again normalized by Trimmed Mean of M-values (TMM) using the *calcNormFactors* function via the edgeR package^23^. The *voom*-transformed data were then used to derive final DE gene list via the *limma* package^21^. As previously described, the PCA plot was generated for this preprocessed TCGA data to visualize a clear separation according to the disease status.

**Figure 3:**
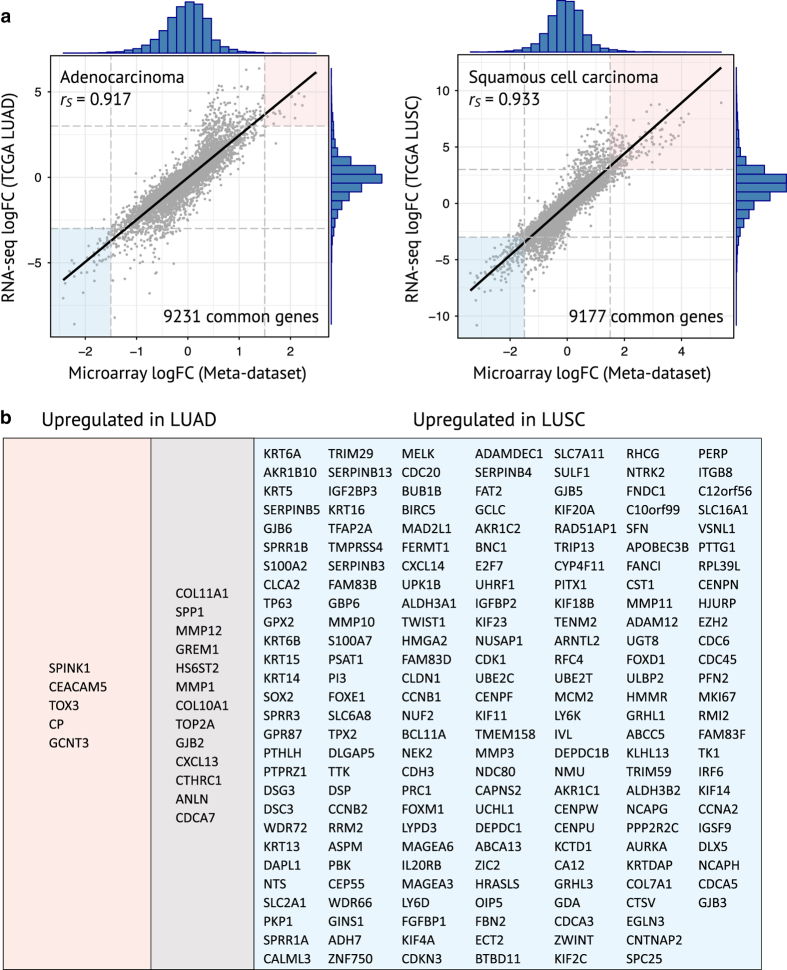
The interplatform concordance between microarray (normalized dataset) and RNA-Seq (TCGA) platforms in discovering DE genes for distinct subtypes of NSCLC. (**a**) Linear regression lines (black line) and marginal histograms (blue) are drawn; *rs*=Spearman’s correlation coefficient. (**b**) DEG lists generated for adenocarcinoma and squamous cell carcinoma (SCC). logFC >1.5 and logFC >3 were used for statistical criteria to define DE genes for our normalized dataset and TCGA cohorts, respectively.

To further demonstrate the utility of our generated dataset in identifying unique set of genes defining distinct subtypes of NSCLC, we performed separate meta-analyses of adenocarcinoma and squamous cell carcinoma (SCC). DE gene lists obtained from the two subtypes were then compared with that from TCGA LUAD and LUSC cohorts, respectively (Fig. 3a). To dispel any bias that could be introduced from different number of genes assayed within each platform, only common genes included in the final DE gene lists were ranked and compared. Regardless of cancer subtypes, a high degree of overlap between DE genes derived from the two platforms was observed (Spearman’s correlation coefficient *r*_*s*_=0.917 and 0.933 for ADC and SCC, respectively). We further identified uniquely and commonly up-regulated DE genes in tumors compared to control tissues (Fig. 3b) by applying our defined cutoff thresholds (logFC >1.5 and logFC >3 for the microarray-based dataset and RNA-seq-based TCGA dataset, respectively).

The present normalized dataset of lung cancer together with its associated clinical metadata will allow exploration of distinct patterns of DE genes in relation to clinical features, including histology, gender, age, pathological and TNM stage, and survival outcomes, facilitating clinical predictive modeling for accurate diagnosis and prognosis in oncology.

## Additional information

**How to cite this article**: Lim, S. B. *et al.* A merged lung cancer transcriptome dataset for clinical predictive modeling. *Sci. Data* 5:180136 doi: 10.1084/sdata.2018.136 (2018).

**Publisher’s note**: Springer Nature remains neutral with regard to jurisdictional claims in published maps and institutional affiliations.

## Supplementary Material



## Figures and Tables

**Table 1:** GSE accession number and number of samples for each phenotype.

	Dataset	Lung tissue	Microarray	Platform
1	GSE10799	3	16	Affymetrix Human Genome U133 Plus 2.0 Array
2	GSE12667	0	75	Affymetrix Human Genome U133 Plus 2.0 Array
3	GSE50081	0	181	Affymetrix Human Genome U133 Plus 2.0 Array
4	GSE31210	20	226	Affymetrix Human Genome U133 Plus 2.0 Array
5	GSE18842	45	46	Affymetrix Human Genome U133 Plus 2.0 Array
6	GSE10445	0	72	Affymetrix Human Genome U133 Plus 2.0 Array
7	GSE33356	60	60	Affymetrix Human Genome U133 Plus 2.0 Array
8	GSE19188	65	91	Affymetrix Human Genome U133 Plus 2.0 Array
9	GSE28571	0	100	Affymetrix Human Genome U133 Plus 2.0 Array
10	GSE10245	0	58	Affymetrix Human Genome U133 Plus 2.0 Array
	*TOTAL*	193	925	1118
